# Donor Sites and Harvesting Techniques Affect miRNA Cargos of Extracellular Vesicles Released by Human Adipose-Derived Mesenchymal Stromal Cells

**DOI:** 10.3390/ijms25126450

**Published:** 2024-06-11

**Authors:** Caterina Visconte, Michela Maria Taiana, Alessandra Colombini, Paola De Luca, Enrico Ragni, Laura de Girolamo

**Affiliations:** IRCCS Istituto Ortopedico Galeazzi, Via R Galeazzi 4, 20161 Milano, Italy; caterina.visconte@grupposandonato.it (C.V.); michelamaria.taiana@grupposandonato.it (M.M.T.); alessandra.colombini@grupposandonato.it (A.C.); deluca.paola@grupposandonato.it (P.D.L.); laura.degirolamo@grupposandonato.it (L.d.G.)

**Keywords:** miRNA, extracellular vesicles, adipose-derived stem cells, adipose tissue, osteoarthritis

## Abstract

Osteoarthritis (OA) is a degenerative joint disorder characterized by the progressive deterioration of articular cartilage driven and sustained by catabolic and inflammatory processes that lead to pain and functional impairment. Adipose-derived stem cells (ASCs) have emerged as a promising therapeutic strategy for OA due to their regenerative potential, which mainly relies on the adaptive release of paracrine molecules that are soluble or encapsulated in extracellular vesicles (EVs). The biological effects of EVs specifically depend on their cargo; in particular, microRNAs (miRNAs) can specifically modulate target cell function through gene expression regulation. This study aimed to investigate the impact of collection site (abdominal vs. peri-trochanteric adipose tissue) and collection method (surgical excision vs. lipoaspiration) on the miRNAs profile in ASC-derived EVs and their potential implications for OA therapy. EV-miRNA cargo profiles from ASCs of different origins were compared. An extensive bioinformatics search through experimentally validated and OA-related targets, pathways, and tissues was conducted. Several miRNAs involved in the restoration of cartilage homeostasis and in immunomodulation were identified in all ASC types. However, EV-miRNA expression profiles were affected by both the tissue-harvesting site and procedure, leading to peculiar characteristics for each type. Our results suggest that adipose-tissue-harvesting techniques and the anatomical site of origin influence the therapeutic efficacy of ASC-EVs for tissue-specific regenerative therapies in OA, which warrants further investigation.

## 1. Introduction

Osteoarthritis (OA) is a degenerative joint disorder characterized by the progressive deterioration of articular cartilage, subchondral bone and synovial membrane leading to pain and functional impairment [1]. In particular, the pathogenesis of OA results from a complex biological imbalance between the reparative and destructive fates of joint tissue, involving mechanical, inflammatory and metabolic factors [1]. Indeed, in arthrosic cartilage, chronic degradative and inflammatory processes are sustained by resident cells (chondrocytes and synoviocytes) and infiltrating inflammatory cells (macrophages and T cells) [2]. A growing body of research emphasizes the intricate interplay between inflammatory processes and the development of osteoarthritic changes within the joint. The imbalance between M1 and M2 macrophage subtypes, particularly in the synovium, plays a crucial role in the progression of OA. Additionally, the excessive production of cytokines from the inflamed synovium triggers cartilage degradation by promoting additional release of matrix metalloproteinases (MMPs) and aggrecanases [3]. Moreover, in OA patients, there is a predominance of pro-inflammatory Th1 cells in the synovial infiltrate, further contributing to the inflammatory environment in the affected joints [4]. Currently, pharmacological treatments are not expected to directly influence damaged tissues, but rather to control symptoms by reducing inflammation and pain [5]. New, promising, biological cell-based products have recently emerged, among which mesenchymal stromal/stromal cells (MSCs) have gained great interest mainly due to their well-established pro-regenerative properties and anti-inflammatory effects [6,7,8], representing an attractive therapeutic strategy for the treatment of OA [9]. Indeed, they have already been used in some clinical trials to treat OA patients for their immunomodulatory and trophic properties, potentially acting on multiple pathological targets by paracrine actions [10]. Among the different sources of MSCs, adipose tissue serves as an abundant and easily accessible one. Adipose-derived stem cells (ASCs) adaptively release several soluble factors and extracellular vesicles (EVs) containing a variety of bioactive molecules, mainly miRNAs. MiRNAs are small, non-coding RNAs that can degrade or block the translation of target mRNAs, resulting in the downregulation of corresponding proteins. The MiRNAs in OA are widely involved in mechanisms associated to inflammation-mediated progression, as well as to different cellular processes, including the protection of cartilage and synovium [11,12]. The biological effects of EVs critically depend on their specific miRNA cargos, due to their crucial role in gene expression regulation. 

It has been observed that ASCs harvested from different anatomical areas exhibit different characteristics with regard to cell counts, viability, gene expression profiles, and function, and also that different harvesting procedures may affect ASC quality, functionality, and plasticity [13]. However, a direct comparison of the miRNAs embedded in the EVs of differently harvested ASCs is still lacking. A clear understanding of how collection sites and harvesting methodologies can also affect the miRNA profile is necessary to optimize the therapeutic efficacy of ASC-derived EVs in OA. The present study aimed to analyze the composition of miRNA cargos of EVs released by different human ASCs to investigate the possible impact of the site and the methodology of adipose tissue-harvesting. The miRNA content of ASC-EVs isolated from adipose tissue was obtained through direct excision of peri-trochanteric (hip) adipose tissue (H-ASC) or from abdominal adipose tissue, either excised surgically (A-ASC) or by lipoaspiration (LipoA-ASC), using identical experimental approaches and platforms [14,15]. MiRNA expression profiles were analyzed by bioinformatic tools for miRNA–mRNA interactions using expression data from OA-affected tissues and cells. 

## 2. Results

### 2.1. Phenotype Characterization of ASCs and Secreted EVs

A total of 13 OA patients were recruited at the IRCCS Ospedale Galeazzi Sant’Ambrogio (Milan, Italy). Three patients (two female and one male) underwent total hip arthroplasty, while five female patients underwent abdominal liposuction. In addition, subcutaneous adipose tissue from local abdominal fat was surgically obtained from five female patients. Samples were collected from patients not presenting with active oncological, rheumatological or metabolic diseases. Demographic information for all participants is summarized in Table S1. Donors from the A-ASC group had an overall younger age than the other two groups, whereas no difference was observed in terms of the bone mass index (BMI) among groups. 

**Figure 1:**
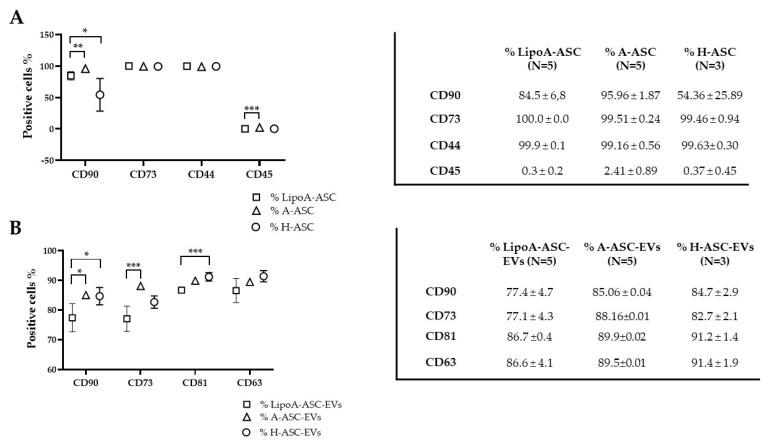
ASC immunophenotype and EV characterization: (**A**) immunophenotype of A-ASC, LipoA-ASC and H-ASC as a percentage of positivity for MSC (CD44/CD73 and CD90) and hematoendothelial (CD45) markers. Mean ± SD, *p* < 0.05, *; *p* < 0.005, **; *p* < 0.001, ***; and (**B**) percentage of positivity for selected markers in A-ASC-EVs, LipoA-ASC-EVs and H-ASC-EVs, showing the presence of EV-defining molecules CD63 and CD81. EVs were also strongly positive for stromal markers CD73 and CD90 labeling. Mean ± SD, *p* < 0.05, *; *p* < 0.005, **; *p* < 0.001, ***.

ASCs were characterized by flow cytometry for MSC and hematoendothelial markers. As shown in Figure 1A, all ASC types were strongly positive for stromal CD44-73-90 and negative for CD45, as previously reported [16]. The lower CD90 expression in H-ASC with respect to the other two ASC types could be explained by the reduction of this marker expression with passages in culture, as already reported [17]. The ASCs showed a similar proliferation rate and population-doubling time, with a longer population-doubling time for A-ASC compared to LipoA-ASC group, although this was not significant (Table S2). To detect particles within the nanometer range, the flow cytometer was calibrated with FITC-fluorescent beads of a predetermined size (from 100 to 900 nm). Purified EVs were strongly positive for EV markers CD63 and CD81. In addition, MSC markers CD73 and CD90 were also highly expressed [18] (Figure 1B).

### 2.2. Comparison of LipoA-ASC-, A-ASC- and H-ASC-Derived Ev-miRNA Profile

**Figure 2:**
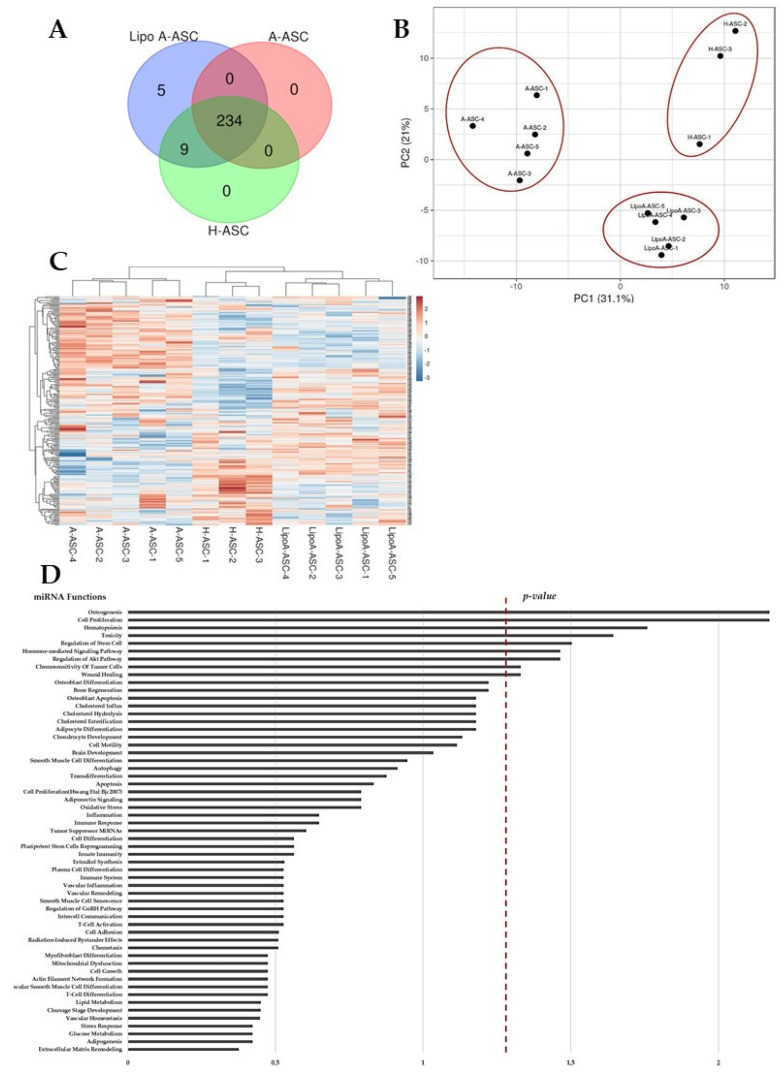
Comparison of EV-derived miRNAs in A-ASC-EVs, LipoA-ASC and H-ASC-EVs: (**A**) Venn diagram showing the distribution of all detectable miRNAs found in EVs from all ASC types; (**B**) principal component analysis (PCA) based on miRNA expression in EVs from A-ASC, LipoA-ASC and H-ASC; (**C**) hierarchical clustering of all ASC types based on miRNA profiles observed in EVs. The most variant (according to the coefficient of variation) miRNAs were used for clustering analysis (Pearson correlation, average linkage). Rows are centered and unit variance scaling is applied to normalized expression values. Color indicates relative up- (red) or downregulation (blue) for each miRNA (row); and (**D**) MiRNA pathway prediction. Biological miRNA functions associated with first-quartile miRNA signatures common in all ASC-EV types. The significance of this association is expressed with –log10 (*p*-value).

MiRNAs in ASC-EVs were profiled with RT-qPCR using the TaqMan Open Array miRNA panel, which enables the testing of 754 human miRNAs. MiRNAs that were detectable in all samples for each ASC-EV type were searched for. In total, 234, 243, and 248 miRNAs were detected and highly shared in A-ASC-EVs, H-ASC-EVs and LipoA-ASC-EVs (Figure 2A). The LipoA-ASC type exclusively expressed five miRNAs (miR-1305, miR-541-3p, miR-376a-5p, miR-941, miR-100-3p). Furthermore, nine miRNAs (miR-373-3p, miR-576-5p, miR-648, miR-1179, miR-566, miR-135b-3p, miR-548a-3p, miR-765, miR-501-5p) were commonly present in both LipoA-ASC-EVs and H-ASC-EVs. 

Although most miRNAs are shared between EVs, principal component analysis (PCA) and hierarchical clustering were able to strongly cluster and group the EV types, showing a greater difference in A-ASC compared to the other two ASC types (Figure 2B). These results were confirmed by inter-condition correlation analysis, where A-ASC samples had low R2 values when compared to LipoA-ASC (0.48) or H-ASC (0.38). The heat map clustering also revealed a proximity between the H-ASC and LipoA-ASC types, which can be explained by the sharing of a number of miRNAs, as shown above (Figure 2C).

For further analysis, we considered miRNAs that were in the first quartile of expression for each ASC-EVs type to concentrate our statistical analyses on the most reliable miRNAs. To give an overview of the roles of the most abundant miRNAs shared between ASC types, target prediction analysis was performed. Figure 2D shows the biological functions associated with first-quartile miRNAs. The majority of the miRNAs were associated with inflammation and immune responses, as well as osteogenesis, adipocyte differentiation, and stem cell regulation.

### 2.3. EV-miRNAs Involved in OA Pathological State

Among the 57 miRNAs defining each quartile, we searched for those most abundantly (≥1% genetic weight) expressed. Nine miRNAs were shared between the three populations, whereas 4 were specifically expressed in A-ASC-EVs, 1 in H-ASC-EVs and 1 was shared only between A-ASC-EVs and H-ASC-EVs. The focus was on those miRNAs reported to be directly involved with OA-cartilage pathogenesis. Albeit inter-groups heterogeneity, one degenerative, and six protective, miRNAs were identified in all the ASC types. Scoring EV-miRNA abundance, as a whole, cartilage-protective miRNAs exceeded cartilage-destructive miRNAs in all groups. In particular, the protective vs. destructive ratio was 5.7 for LipoA-ASC, 2.8 for A-ASC, and 8 for H-ASC. Notably, two miRNAs associated with overlapping roles in OA cartilage homeostasis were present in all ASC groups, whereas two miRNAs were shared only between LipoA-ASC and H-ASC. Additionally, miR191-5p was the unique miRNA involved in synovial regulation in LipoA-ASC and H-ASC (Table 1).

Regarding immunomodulation, three miRNAs involved in macrophage switch (M1 pro-inflammatory to M2 anti-inflammatory) were present in the ASC-EVs cargo. Overall, influence on the M2 phenotype was more pronounced, with a pro-M2 vs. pro-M1 miRNAs ratio of 13.7 for LipoA-ASC, 2.7 for A-ASC and 19.2 for H-ASC. In particular, the most abundant miRNA identified in all types was miR-24-3p, which is responsible for M2 differentiation by blocking M1 activation (Table 1).

Among the miRNAs known to be involved in T-cell immunoregulation, all three ASC types shared a higher abundance for T-cell activation inhibitors compared to T-cell activation mediators (with a ratio of 7.4 for LipoA-ASC, 5.9 for A-ASC, and 4.4 for H-ASC). In particular, four miRNAs involved in the negative regulation of T-cell activation were identified, with miR-24-3p and miR-125b-5p being the most represented in all ASC types. For T-cell activation, miR-221-3p was the most abundant in all the ASCs analyzed (Table 1).

The miRNA differential expression levels were further analyzed by using the 2^−ΔCT^ method (Table 2). This analysis confirmed distinct miRNAs expression profiles in EVs released from ASCs isolated from the abdomen and hip. 

A comparison between LipoA-ASC and A-ASC showed five downregulated and eight upregulated miRNAs, with the most reduced being miR-145-5p, and the most induced being miR-30c-5p (Table 2).

Comparing A-ASC to H-ASC, eight downregulated and six upregulated miRNAs were observed, where miR-320a-3p and miR-21-5p were the most reduced and induced, respectively.

Comparison between LipoA-ASC vs. H-ASC groups found only one downregulated miRNA, mir-320a-3p, whereas five miRNAs were upregulated, with miR-100-5p being the most increased.

### 2.4. EV-miRNA Target Identification

After sifting experimentally validated miRNA–mRNA interactions, the most abundantly expressed miRNAs targeted 4856 univocal genes in LipoA-ASC, 4184 genes in A-ASC, and 5199 genes in H-ASC. To evaluate the impact of ASC-EV miRNAs in the context of OA, validated targets of the most abundant miRNAs with genes known to be involved in OA progression were compared (Table 3). 

**Figure 3:**
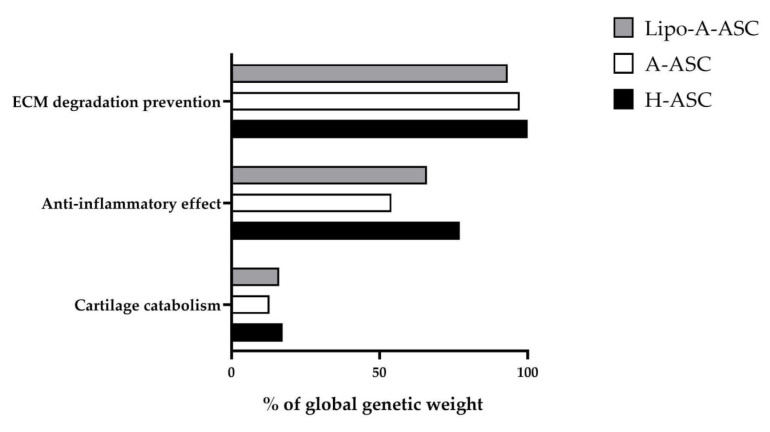
The bar graph displays the contribution, expressed as a percentage of the total genetic weight of all miRNAs involved in regulating three of the primary mechanisms associated with OA.

Total miRNA genetic weight for each targeted transcript was obtained, considering each miRNA contribution. Genes involved in ECM homeostasis and expressed in both chondrocytes and synoviocytes were the main targets of all EV-miRNA types, with *MMP2/13/14*, *ADAM16/17*, *APC*, *CTSD* having the strongest regulation (for the main regulators see Table 2 and Table 3). LipoA-ASC resulted in having a lower global genetic weight associated with proteases and activators involved in ECM degradation compared to A-ASC and H-ASC (Figure 3). Concurrently, the metalloproteases inhibitor *TIMP3* was targeted by EV-miRNAs, being regulated mainly by miR-222-3p in LipoA-ASC and H-ASC, and by miR-21-5p in A-ASC. In line with these data, the analysis of miRNA expression revealed significant downregulation of miR-222-3p and upregulation of miR-21-5p in A-ASC-EVs compared to the other two groups (Table 2). miR-222-3p also regulated metalloproteases inhibitor *TIMP2* in all EV-ASC types. 

Several other miRNA-targeting genes related to cartilage catabolism were consistently present in all ASC-EV types, with a lower total genetic weight for A-ASC (Figure 3). 

*LIF*, *CXCL12*, *CSF1* were shown to be the most heavily targeted transcripts mainly by respectively miR-125b-5p (equally expressed in all types), miR-221-3p (downregulated in A-ASC), and miR-30c-5p (downregulated in A-ASC) (Table 2).

The H-ASC-EV type presented the highest global genetic weight of miRNA-targeting OA-related inflammatory mediators (Figure 3). Among these factors, TNF emerged as the most heavily targeted transcript, being regulated by three different miRNAs with a relevant genetic weight in all the ASC-EV types, namely miR-24-3p, miR-100-5p and miR-125b-5p. The main contributor was miR-24-3p, which was found to be significantly downregulated in A-ASC compared to the other two groups. Two other M1 inflammatory cytokines, *IL-1β* and *IL-6* [19], were also targeted by EV-miRNA, suggesting a preponderance for M2-resolving macrophage polarization, which was again more relevant for H-ASCs. These factors were, respectively, regulated by miR-21-5p, which was significantly upregulated in the A-ASC-EV type, and miR-125b-5p, which was equally expressed in all ASC-EV types (Table 2). Other inflammatory molecules expressed in synovial macrophages, such as *IFNγ*, *C5*, *IL1A*, *IL11*, and *IL18*, were heavily targeted by EV-miRNAs present in all three types (for main regulators see Table S3 and Table 2). Importantly, the anti-inflammatory *IL4* molecule was also regulated by miR-24-3p in all ASC-EVs.

miR-24-3p was the main regulator of growth factors as the destructive molecule *TGFB1* and the abnormal angiogenesis promoter *ANGPT2*. *VEGFA*, also known to be involved in angiogenesis, was mainly targeted by miR-221-3p in LipoA-ASC and H-ASC, and by miR-21-5p in A-ASC. EV-miRNAs also targeted a few protective molecules, such as *IGF1* and *IGF2*, being the target, respectively of miR-24-3p and miR-125b-5p, which also regulated *IL1RN*, a suppressor of cytokine-induced catabolism in cartilage [20]. 

### 2.5. Target Effect Prediction of EV-miRNAs and Comparisons among ASC Types

The analysis of relative quantification (reported as fold change) of miRNAs with genetic weight ≥ 1 (Table 2) led to the identification of principal deregulated miRNAs between groups (fold > 2 or <0.5, adjusted *p*-value ≤ 0.05). 

LipoA-ASC-EVs compared to A-ASC-EVs had five upregulated and three downregulated miRNAs. Overall, the deregulated miRNAs targeted genes associated with the inflammatory processes and with the negative regulation of ECM degradation mechanisms. Specifically, upregulated miRNAs are predicted to negatively regulate genes with proinflammatory involvement as *IL11*, *IL1A* and *KITLG*, likely leading to a higher anti-inflammatory effect of LipoA-ASC-EVs compared to A-ASC-EVs and the metalloprotease inhibitors *TIMP2* and *TIMP3*. The downregulated miRNAs targeting genes involved in ECM degradation promote the expression of genes as metalloproteinases *MMP1/2/9/14,* and angiogenic factors *ANGPT2* and *VEGFA*, as well as growth factors *TGFB1/2*.

A-ASC-EVs, compared to H-ASC-EVs, had five upregulated and four downregulated miRNAs. Upregulated miRNAs are predicted to decrease the expression on genes mainly associated with chondrocyte cartilage catabolic activity and ECM degradation, including *MMP1/2/9/13/14*, *TGFB1/2*, *ADAM14*, *PLAT* and *APC*, several growth factors, such as *EGF*, *FGF1*, *HGF*, CTG, and angiogenic factors *ANGPT2* and *VEGFA.* Moreover, *TIMP2* and *3* are predicted to be upregulated by decreased miRNA. Genes associated with inflammatory pathways, such as *IL1A*, *IL11*, and *KITLG* are predicted to be more highly expressed as result of the effect of downregulated miRNA. However, the lower anti-inflammatory effect could be counterbalanced by the decrease in the expression of proinflammatory genes as a result of the increase in the miRNAs for which they are targeted. Finally, only four miRNAs were upregulated in LipoA-ASC-EVs compared to H-ASC-EVs. These miRNAs are predicted to negatively regulate the mechanisms associated with cartilage degradation by acting on several target genes, such as *MMP2/9/1/13*, *PLAT*, *APC* and *TGFB1/2*, and *VEGFA* and *WNT1*, attenuating their negative effect on OA progression; only the positive factor MMP inhibitor *TIMP3* is predicted to be downregulated.

## 3. Discussion

The main findings of the study show that, as part of an overall shared molecular signature targeting various factors and processes related to OA, all ASC types were able to release EVs carrying miRNAs with anti-inflammatory and cartilage-protective properties. However, analysis revealed distinct miRNA expression profiles between the different types, suggesting potential variability in their therapeutic efficacy on OA joints. 

The effectiveness of MSCs in repairing cartilage and alleviating the symptoms of OA has been demonstrated in recent studies [21,22]. Through their paracrine activity, MSCs are able to influence the environment and mediate endogenous tissue repair, mainly by secreting soluble bioactive factors encapsulated in EV [23,24]. Indeed, EVs were recently proposed as standalone cell-free therapeutic agents for several pathologies, including OA [25,26]. 

ASCs have emerged as a highly promising MSC population due to their widespread presence and relatively simple harvesting process, which results in less donor-site morbidity and allows for larger quantities to be obtained. Subcutaneous adipose tissue is considered the most clinically relevant source of ASCs and it can be easily isolated from multiple anatomical sites such as the abdomen, peri-trochanteric area, thigh, and arm [13]. Several studies have reported significant inter-individual heterogeneity in adipose tissue features particularly linked to body mass index [27], age [28] and sex [29]. Furthermore, intra-individual differences in fat depots have been also observed in the in histological features, such as vascularity and the amounts of fibrous tissue. Differences were also observed in the cellular composition of adipose tissue, in particular in ASC counts and viability, but also in function and in the way in which adipose tissue responds metabolically to hormonal and neurological stimuli [30]. In addition to the anatomical site of harvesting, another controversial factor concerns the procedure for ASC collection that allows for the best cell yield, viability, proliferative index and stemness [31,32]; the standard protocol for harvesting adipose tissue for ASCs is yet to be defined. Indeed, some specific technical aspects of the different procedures can influence ASCs properties. Infiltration fluid and mechanical shearing forces applied for the harvesting of adipose tissue in tumescent liposuction could potentially damage the stromal vascular fraction cells, affecting ASC viability. Moreover, irradiation with high-intensity ultrasonic energy during ultrasound-assisted liposuction combined with localized temperature elevation and mechanical stress could lead to the production of radicals and oxidizing agents and DNA damage with consequent long-term effects on multiple cellular pathways [31,32]. This study showed that the expression profile of miRNAs embedded in EVs released from ASCs is influenced by both the anatomical site and the procedure of adipose-tissue-harvesting. In fact, donors within the same ASC type were more homogeneous compared to donors of other types, suggesting that the effect of different procedures and harvest sites outweighs the biological variability between donors in terms of the differences in miRNA expression, allowing the expressions to sharply cluster the different groups. Our donors showed homogeneity with regard to sex, where only one was male. In contrast, donors belonging to the A-ASC group were younger overall than donors in the other two groups. However, despite this difference, clustering based on miRNA expression revealed a clear molecular separation on harvest procedure and anatomical site rather than on age. 

In order to attribute a biological significance to the detected or differentially expressed EV-miRNAs, miRNAs laying in the first quartile of expression and with higher genetic weights in all ASC-EV groups were particularly addressed. The majority of miNAs showed consistent expression across different ASC types and were mainly involved in inflammatory pathways and ECM homeostasis, highlighting their potential therapeutic relevance in OA treatment. Global genetic weight analysis pointed out that H-ASC-EVs possess the strongest involvement in the regulation of proinflammatory pathways, whereas A-ASC-EVs had the lowest. The same result was obtained for the cartilage catabolism pathway, although with a smaller difference between the groups. Again, H-ASC-EVs showed the highest weight for the ECM degradation pathway. Regarding other genes mainly related to OA progression and acting in different pathways, including angiogenesis, A-ASC-EVs seemed to have the highest genetic weight. 

The gene with the heaviest regulation in all the ASC types was the proinflammatory gene *TNF*, whose inhibition was reported to have some therapeutic effects in OA [33]. The main regulator of *TNF* was miR-24-3p in all three cell types. MiR-24-3p resulted to be one of the most-expressed miRNAs in ASC-EV cargos presented in all the types, likely representing one of the prevalent players of the potential therapeutic effect of EVs in OA. Interestingly, miR-24-3p was reported to be downregulated in OA progression, suggesting a beneficial effect of the restoration of its expression level [34]. MiR-24-3p reduces both metalloprotease secretion and positively regulates cartilage catabolism [35], but has a relevant role also in the inflammatory pathway, repressing IFNγ, and inhibiting T-cell proliferation and Th2 differentiation by limiting IL4 production. Moreover, miR-24-3p overexpression also significantly reduces *TNFα* levels, inhibiting M1 polarization and increasing M2 phenotypes [36]. Another predicted target gene for miR-24-3p is *ANGPT2,* responsible for the abnormal angiogenesis in OA with *VEGF* and therefore involved in disease progression, which was shown to be another of the most heavily regulated genes for all EVs groups in our study.

Among the differentially expressed miRNA, miR-145 was significantly more expressed in A-ASC compared to the other ASC-EV types. Interestingly, this miRNA targeted several genes involved in pathways associated with the progression of OA. Studies have shown that miR-145 can reduce chondrocyte apoptosis induced by OA by targeting *BNIP3* and regulating the Notch signaling pathway [37]. Moreover, several genes involved in the expression of the extracellular matrix and inflammation in primary chondrocytes are directly targeted by miR-145 [38]. 

Likewise, miR-21-5p was significantly more abundant in A-ASC-EVs. It was found to be upregulated in OA chondrocytes, acting as a promoter of the progression of the pathology by targeting *GDF-5*, a signal for chondrogenesis that promotes chondrocyte differentiation [39,40,41]. On the other hand, miR-21-5p is likely to have an anti-inflammatory role, since it has been shown to inhibit *IFNγ* and T-cell activation and to positively regulate IL-10 secretion [42]. These studies suggest that miR-21-5p may be a novel target for the treatment of patients with OA [41]. In A-ASC-EVs, miR-320a was also upregulated and has been reported in the literature to be important in the regulation of OA, involving the ERK/JNK/MAPK pathway. Indeed, in vitro experiments demonstrated that overexpression of miR-320a restored chondrocyte proliferation inhibited by IL-1β, and reduced apoptosis and inflammatory response [43].

MiR-30b-5p and miR-30c-5p were instead downregulated in A-ASCs compared to the other ASCs. Growing evidence suggests that the miR-30 family may be involved in initiating and developing joint diseases. MiR-30b-5p was found to be upregulated in cartilage tissue of OA patients, correlated to progressive stages of OA and enhanced levels of pro-inflammatory mediators in the synovial fluid. MiR-30c-5p in OA synovial fluid was shown to correlate with clinical symptoms and was named as a possible therapeutic target [44].

MiR-222-3p is one of the miRNAs responsible for cartilage degradation in human OA targeting MMP-13. In vivo studies showed that over-expression of miR-222 significantly reduced cartilage destruction in mice model [45]. MiR-222-3p was shown to be less expressed in A-ASCs, whereas miR-99a-5p was shown to be reduced in LipoA-ASC compared to the other types. MiR-99a-5p has been shown to affect macrophage polarization [46] and was shown to be downregulated in OA lesions [11]. MiR-100-5p was significantly upregulated in both A-ASC and LipoA-ASC compared to H-ASC. Preclinical studies showed that miR-100-5p mediated inhibition of mTOR-autophagy pathway in the maintenance of articular cartilage [47]. 

**Figure 4:**
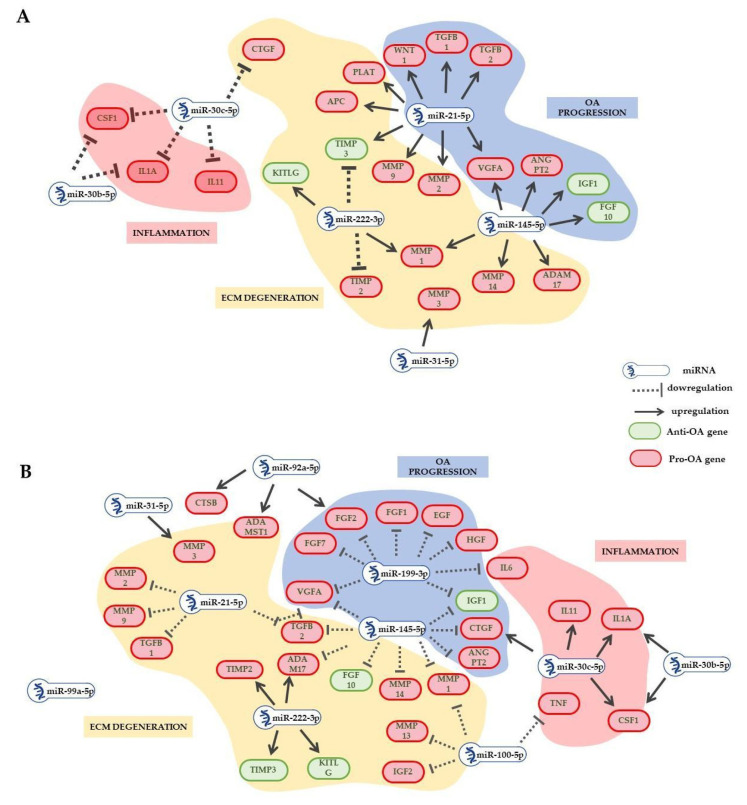
LipoA-ASC-EVs vs. A-ASC-EVs: (**A**) and A-ASC-EVs vs. H-ASC-EVs; and (**B**) predicted target regulation. Red circles are pro-OA genes, green circles are anti-OA genes. Black arrows showed induced expression, dashed blocking arrows showed inhibited expression.

Considering globally genes predicted to be targeted by significantly differential expressed miRNAs, the main differences between LipoA-ASC-EVs and A-ASC-EVs were likely to be related to ECM homeostasis. Taken as a whole, A-ASC-EVs expressed higher levels of miRNAs involved in ECM degradation, blocking the expression of several ECM-degrading proteins more effectively than LipoA-ASC-EVs (Figure 4A). Notably, A-ASC miRNAs expression profile is predicted to block other genes involved in OA progression such as *ANGPT2*, *VEGF2* and *WNT1*. On the other hand, LipoA-ASC-EVs seem to decrease expression of a higher number of proinflammatory molecules compared to A-ASC-EVs. Regarding ECM and cartilage protection, numerous genes involved in ECM degradation and cartilage catabolism were predicted to be downregulated by highly expressed miRNAs in A-ASC-EVs (Figure 4B) compared to H-ASC-EVs. *ANGPT2*, *VEGF2* and *WNT1* were also likely to be downregulated in A-ASC-EVs in comparison to H-ASC-EVs. The effect on inflammation process cannot be clearly assigned, as different inflammatory molecules were predicted to be negatively or positively regulated.

Previous studies have pointed to adipose-tissue-harvesting from the abdomen through direct excision as the ideal method to maximize the yields of ASCs [29,48]. However, others have hypothesized the advantage of lipoaspiration, especially because of the less invasive nature of the procedure and the higher percentage of viable cells, along with a similar adipogenic differentiation potential between lipoaspirated and resected ASCs [31]. In the present study, computational analysis identified distinct miRNA expression profiles between A-ASC-EVs, LipoA-ASC-EVs, and H-ASC-EVs, suggesting potential differences in their therapeutic effectiveness on OA joint variability. To complete this picture, a comparison between different strategies for harvesting adipose tissue from the hip would be valuable to confirm the impact of the harvesting technique on a specific anatomical site. 

One significant limitation of this study is the lack of experimental validation of the computational data. Indeed, functional validation is necessary to confirm the therapeutic superiority of one procedure over others. 

A single miRNA can regulate multiple genes, and one gene can be regulated by multiple miRNAs; the complexity of miRNA-target networks is too great to be clearly understood [49]. The continuous improvement of experimental strategies has further revealed the complexity of the mechanisms of miRNA–target interaction, highlighting the influence of cell type and cell state and thus identifying new specific targets [49]. Prediction of the global influence of miRNA deregulation is challenging: each single miRNA has a different regulatory effectiveness and the target genes vary in importance in a specific context. Thus, precise miRNAs may have a critical impact on the entire regulatory network for a given pathology [50]. Therefore, further studies in both in vitro and in vivo models of OA are necessary to define the contribution of miRNA dysregulation in OA pathogenesis and progression and to determine the real therapeutic superiority of one procedure over the others. Another limitation is the different age of patients in the A-ASC group compared with the other two groups. The older age of patients in the Lipo-A-ASC and H-ASC groups is related to the reason these patients underwent surgery—and had their waste adipose tissue available for research purposes—namely osteoarthritis, a condition that typically affects older patients. In contrast, patients in the other group, A-ASC, underwent surgery to remove excess skin and fat from the middle and lower abdomen, a procedure typical of patients of all ages, including younger patients. Nevertheless, MiRNA-based clustering did not show separation based on age but rather on the type of procedure and the site of removal. 

## 4. Materials and Methods

### 4.1. hASC Isolation and Culture

Collection of abdominal adipose tissue by lipoaspiration and peri-trochanteric adipose tissue by surgical excision were previously described [14,15]. In these two patient groups, adipose tissue was collected as waste material during regenerative medicine procedures based on fat-derived products and during total hip arthroplasty, respectively. Excised abdominal adipose tissue was obtained as waste material from five donors who underwent abdominoplasty (Table S1). All the donors involved in this study gave their consent to donate waste biological material for research purposes. ASCs were isolated as previously described [19]. Briefly, adipose tissue was digested for 40 min at 37 °C with type I collagenase 2 mg/mL (Worthington Biochemical Co., Lakewood, NJ, USA) and filtered with a 100 µm cell strainer. Cells were recovered by centrifugation (1000× *g*, 5 min), pellets were suspended in α-MEM or DMEM high glucose (Sigma Aldrich, St. Louis, MO, USA) supplemented with FBS (GE Healthcare, Piscataway, NJ, USA) and 1% PSG (ThermoFisher, Waltham, MA, USA) and seeded at 10 × 10^3^ cells/cm^2^. After selection for plastic adhesion, cells were expanded at 37 °C in a humidified atmosphere with 5% CO_2_ and used at Passage 3. Proliferation rate and population-doubling time of ASCs was evaluated from second passage. Cells were detached from the culture flask, counted and population-doubling time (dt) was calculated according to the following formula: tdT = [ ln(2)/μ] × 24 h, where μ is the growth rate of cells and is calculated with the formula: μ = [(ln (Nt/No))/Δt] × 24 h, where No is the number of cells seeded, Nt is the number of cells harvested and Δt are the hours of growth.

### 4.2. hASC Characterization by Flow Cytometry

ASCs from an abdomen in suspension at Passage 3 were stained for 30 min at 4 °C in the dark with the following antibodies and dilutions: 1:20 anti-CD44-APC (REA690, Miltenyi Biotec, Bergisch Gladbach, Germany), 1:50 CD73-PE (REA804, Miltenyi), 1:50 CD90-FITC (REA897, Miltenyi), and 1:50 CD45-PE-Vio770 (REA747, Miltenyi), according to the manufacturer’s instructions. After the cell wash with FACS buffer (phosphate buffer, 5% FBS, 0.1% sodium azide), cells were detected by flow cytometry using a CytoFLEX flow cytometer (Beckman Coulter, Fullerton, CA, USA), collecting at least 20,000 events. Unstained samples (without antibodies) were used as negative controls for background evaluation in FITC, PE and APC channels. For ASCs derived from peri-trochanteric adipose tissue excision, we followed previously described protocols assessed at Passage 4 and 3, respectively [14,51].

### 4.3. hASC Culture Supernatant Collection and EVs Isolation

At 90% cell confluence, T25 culture flasks were washed three times with PBS to remove residues of exhausted media, and 5mL fresh DMEM without supplements were added. After 48 h, media were collected and filtered with a 0.22 μm pore-size filter to eliminate debris, floating cells, and apoptotic bodies. The filtered culture supernatant was further centrifuged at 100,000× *g* for 3 h at 4 °C in a 70.1Ti rotor (Beckman Coulter, Fullerton, CA, USA). Pellets were suspended in PBS (500 μL).

### 4.4. EVs Characterization by Flow Cytometry

EVs were incubated with either PBS or PBS + 0.1 μM CFSE, in the dark at 37 °C for 1 h. Additionally, CFSE-labeled samples were concomitantly stained with 1 μL of the following APC-conjugated antibodies: CD63 (353007, BioLegend, San Diego, CA, USA), CD81 (349509, BioLegend), anti-CD44 (338805, BioLegend), CD73 (344005, BioLegend), and CD90 (328113, BioLegend). After incubation, PBS to a final volume of 150 μL was added to both stained (CFSE and CFSE + Abs) and unstained samples, and events collection was performed with a CytoFLEX flow cytometer at 60 μL/min flow rate. The flow cytometer was previously calibrated with Megamix-Plus SSC and FSC reference beads (Biocytex, Marseille, France) consisting of FITC fluorescent spheres (100, 160, 200, 240, 300, 500, 900 nm). To identify CFSE-positive EVs, a first gate in the FITC channel was performed using unstained EVs as negative samples. FITC + events were used to create APC positive and negative gates to visualize the EVs carrying the respective antigens in CFSE + Abs samples. 

### 4.5. EV-miRNA Cargo Evaluation and Normalization

EV-miRNA qRT-PCR data were obtained, as previously published [18]. Briefly, TaqMan^®^ MicroRNA Reverse Transcription Kit and related Megaplex™ RT Primers were used to synthesize single-stranded cDNA from the small RNA samples. For a full miRNA profile, we performed two separate reverse transcription reactions for each sample using pool A or pool B Megaplex^TM^ RT primers, followed by RT-PCR analysis with the QuantStudio^TM^ 12 K Flex OpenArray^®^ Platform on A and B miRNA panels, which together cover 754 human miRNA sequences from the Sanger miRBase v21.

MiRNA expression stability was evaluated by adding nonhuman synthetic miRNA spike-in (Arabidopsis thaliana ath-miR-159a). Subsequently, only miRNAs present in all five ASCs and shared among the three ASC groups (A-ASC-EVs; lipo A-ASC-EV and H-ASC-EVs) were considered for normalization, performed with a global mean strategy [10]. In addition, only miRNAs in the first quartile of expression of all ASC-EV types were considered for further analysis, covering approximately 88% of the genetic message. The genetic weight was calculated using the ΔCt method between normalized miRNAs (using the lowest normalized Ct as a reference point for ΔCt calculations between miRNAs), assigning an arbitrary value of 1 to the lowest normalized Ct and 2^−ΔCt^ value.

### 4.6. EV-miRNA Target Identification

The miRTarBase database https://mirtarbase.cuhk.edu.cn/~miRTarBase/miRTarBase_2022/php/index.php (accessed on 15 September 2019) was used to retrieve the EV-miRNA targets, selecting only those interactions reported to be validated by strong experimental evidence (reporter assay, Western blot, and qPCR). 

To provide visual exploration and functional interpretation of the miRNA–target interaction network and a pathway enrichment analysis, we used MiRNet (https://www.mirnet.ca/miRNet/home.xhtml, accessed on 20 July 2023) web tool [52,53]. 

### 4.7. Statistical Analysis

Normalized Ct values of miRNAs were used to analyze differences between ASC groups. The statistical analysis was performed using GraphPad Prism 8.0 Software (GraphPad Software Inc., San Diego, CA, USA). The data distribution was tested for normality with Kolmogorov–Smirnov and Shapiro–Wilk tests. Multiple comparisons were performed using a one-way ANOVA followed by Tukey’s test or the Kruskal–Wallis test followed by Dunn’s Multiple Comparison test as post hoc tests. The Spearman test was applied for correlations between Ct values of deregulated miRNAs between ASC types.

## 5. Conclusions

This report underscores the importance of considering adipose-tissue-harvesting techniques and anatomical sites of origin in optimizing the therapeutic potential of ASC-EVs for tissue-specific regenerative therapies in OA, and lays the groundwork for future comparative functional and clinical studies to validate the efficacy of specific ASC-EV types in OA treatment.

## Figures and Tables

**Table 1:** miRNAs with genetic weight ≥ 1 in LipoA-ASC, A-ASC-EVs and HASC-EVs, involved in biological functions related to OA.

	LipoA-ASC-EVs Genetic Weight %	A-ASC-EVs Genetic Weight %	H-ASC-EVs Genetic Weight %	Function
**Cartilage protection**				
miR-24-3p	14.221	10.436	19.180	Regulates chondrocyte senescence.
miR-125b-5p	11.061	8.598	10.468	Prevents aggrecan loss.
miR-193b-3p	3.533	3.782	4.000	Regulates inflammation by repressing TNF-α expression.
miR-100-5p	2.763	3.693	1.005	Inhibition of mTOR-autophagy pathway.
miR-99a-5p	2.714	3.243	1.016	Suppresses cell apoptosis and extracellular matrix degradation stimulated by IL-6 and
miR-222-3p	4.232	2.016	5.009	Reduces cartilage degradation.
miR-320a-3p		1.271		Chondrocyte viability
miR-199a-3p		1.186		Anti-catabolic.
miR-92a-3p	1.261		1.892	Anti-catabolic and increases collagen deposition.
miR-574-3p			1.044	Chondrogenesis regulation mediated by SOX9.
**Cartilage destructive**				
miR-21-5p	4.128	9.150	2.053	Negatively regulates chondrogenesis.
miR-19b-3p		1.803	1.307	Induces NF-κB signaling.
miR-214-3p		1.231		Negatively regulates NF-κB signaling pathway.
miR-30b-5p	1.717		2.078	Pro-apoptotic. ECM degradation.
miR-99b-5p	1.177			Activation of NF-κB signaling in chondrocytes.
**Cartilage overlapping**				
miR-145-5p	1.348	4.657	1.258	Chondrocyte proliferation vs. cartilage degradation.
miR-221-3p	4.154	2.525	6.231	Prevents ECM degradation vs. pro-inflammatory.
miR-30c-5p	2.260		2.399	Involved in the bone morphogenetic protein (BMP) signaling pathway.
miR-31-5p	1.112		1.262	Involved in apoptosis and calcification targeting ATF6.
**Synovia overlapping**				
miR-191-5p	1.292		1.545	Hypoxia-induced cell proliferation.
**Pro M1 macrophage**				
miR-145-5p	1.348	4.657	1.258	M1-promoting.
**Pro M2 macrophage**				
miR-24-3p	14.221	10.436	19.180	M2-promoting, M1-suppressing.
miR-222-3p	4.232	2.016	5.009	M2-promoting.
**T cell**				
miR-24-3p	14.221	10.436	19.180	Negative regulation of T cell activation.
miR-21-5p	4.128	9.150	2.053	Negative regulation of T cell activation.
miR-125b-5p	11.061	8.598	10.468	Negative regulation of T cell activation.
miR-145-5p	1.348	4.657	1.258	Negative regulation of T Reg activation.
miR-221-3p	4.154	2.525	6.231	T cell activation.
miR-19b-3p		1.803	1.307	T cell activation.
miR-214-3p		1.231		T cell activation.
miR-636		13.990		Unknown.
miR-520e-3p	2.253	9.266		Unknown.

**Table 2:** Expression levels of selected miRNAs involved in OA in A-ASC-EVs, H-ASC-EVs and LipoA-ASC by real-time PCR. Mean ± SEM, *p* < 0.05, *; *p* < 0.005, **; *p* < 0.001, ***; *p* < 0.00001, ****. ns not significant.

	FC (Lipo A-ASC vs. A-ASC)	FC (Lipo A-ASC vs. H-ASC)	FC (A-ASC vs. H-ASC)	*p* Value (Lipo A-ASC vs. A-ASC)	*p* Value (Lipo A-ASC vs. H-ASC)	*p* Value (A-ASC vs. H-ASC)
**Cartilage protection**						
miR-193b-3p	1.013	0.959	0.946	ns	ns	ns
miR-320a-3p	0.326	0.544	1.671	****	*	*
miR-199a-3p	0.612	1.701	2.778	**	**	****
miR-222-3p	2.27	0.91	0.040	*	ns	*
miR-24-3p	1.478	0.805	0.545	*	ns	**
miR-92a-3p	1.52	0.72	0.47	ns	ns	*
miR-125b-5p	1.39	1.14	0.82	ns	ns	ns
miR-574-3p	0.926	0.736	0.795	ns	ns	ns
miR-100-5p	0.811	2.986	3.681	ns	***	***
miR-99a-5p	0.908	2.900	3.195	ns	***	***
**Cartilage destructive**						
miR-30b-5p	2.583	0.897	0.347	****	ns	****
miR-21-5p	0.489	2.183	4.460	*	*	***
miR-19b-3p	0.811	2.986	3.681	**	ns	ns
miR-99b-5p	2.766	2.373	0.858	***	**	ns
miR-214-3p	0.724	1.026	1.417	ns	ns	ns
**Cartilage overlapping**						
miR-145-5p	0.314	1.164	3.706	**	ns	**
miR-221-3p	1.785	0.72	0.40	*	ns	**
miR-30c-5p	3.803	1.023	0.269	****	ns	****
miR-31-5p	2.139	0.957	0.447	*	ns	*
**Synovia overlapping**						
miR-191-5p	1.519	0.908	0.598	**	ns	**
**Pro M1 macrophage**						
miR-145-5p	0.314	1.164	3.706	**	ns	**
**Pro M2 macrophage**						
miR-24-3p	1.478	0.805	0.545	*	ns	**
miR-222-3p	2.27	0.91	0.040	*	ns	*
**T cell**						
miR-24-3p	1.478	0.805	0.545	*	ns	**
miR-125b-5p	1.39	1.14	0.82	ns	ns	ns
miR-214-3p	0.724	1.026	1.417	ns	ns	ns
miR-21-5p	0.489	2.183	4.460	*	*	***
miR-221-3p	1.785	0.72	0.40	*	ns	**
miR-19b-3p	0.811	2.986	3.681	**	ns	ns
miR-145-5p	0.314	1.164	3.706	**	ns	**
miR-520e-3p	0.264	268.914	1019.750	0.733	0.048	0.016
miR-636	0.018	2.000	111.430	0.056	>0.9999	0.050

**Table 3:** Factors involved in OA pathological state at cartilage (CHO), synovium (SYN), immune cells (HLA-DR, T CELL) levels and genetic weight of all targeting EV-miRNAs. An ‘X’ identifies the cell type involved in factor release.

Factor	Total Genetic Weight (%) of First-Quartile miRNAs			Expressing Cell Type			
	Lipo-A-ASC	A-ASC	H-ASC	CHO	SYN	HLA-DR *	T CELL
**Cytokines**							
*TNF*	28.04	22.73	30.65		X	X	
*IL6*	11.73	9.78	10.90		X	X	
*IL1B*	18.35	19.59	21.23		X	X	
*IL1A*	5.27	2.29	6.02		X	X	
*IFNG*	14.22	10.44	19.18				
*IL4*	14.22	10.44	19.18				
*CSF1*	3.98	1.37	4.48	X	X	X	
*CXCL12*	5.27	3.09	7.49		X	X	
*WNT1*	4.13	9.15	2.05				
*IL18*	14.22	10.44	19.18		X	X	
*EPO*	11.06	8.60	10.47				
*LIF*	11.06	8.60	10.47	X	X	X	
*C5*	3.53	3.78	4.00		X	X	
*CCL5*	0.82	1.23	0.87		X	X	X
*IL11*	2.26	0.64	2.40	X	X	X	
**Growth Factors**							
*TGFB1*	19.92	22.22	23.58	X	X	X	X
*EGF*	0.67	1.19	0.43				
*IGF1*	17.93	19.31	23.04		X	X	
*FGF2*	1.93	2.08	2.32	X	X		
*BMP2*	1.29	0.92	1.55	X	X	X	
*VEGFA*	10.30	17.52	9.97	X	X	X	
*HGF*	0.67	1.19	0.43		X	X	
*ANGPT2*	27.45	24.92	31.78		X	X	
*CTGF*	4.47	7.10	4.96	X	X	X	
*KITLG*	5.10	3.82	6.32	X	X	X	
*TGFB2*	5.48	13.81	3.31	X	X	X	
*FGF10*	1.35	4.66	1.26		X	X	
*FGF7*	0.67	1.19	0.43	X	X	X	
*IGF2*	13.82	12.29	11.47	X	X		
*FGF1*	0.67	1.19	0.43	X	X		
**Proteases and Other**							
*ADAM16*	15.57	15.09	20.44				
*ADAM17*	15.57	15.09	20.44	X	X	X	
*ADAMTS1*	12.32	9.49	12.36	X	X		
*MMP1*	8.34	10.37	7.27		X		
*MMP2*	19.34	20.27	18.75	X	X		
*MMP3*	1.11	0.56	1.26	X	X		
*MMP9*	4.13	9.15	2.05		X	X	
*MMP13*	13.82	12.29	11.47				
*MMP14*	15.57	15.09	20.44	X	X		
*PLAT*	4.13	9.15	2.05	X	X		
*PLAU*	4.40	5.59	5.31		X	X	
*CTSB*	1.26	0.90	1.89	X	X	X	
*CTSD*	14.22	10.44	19.18	X	X	X	
*APC*	15.19	17.75	12.52	X	X		
*TIMP2*	4.23	2.02	5.01	X	X		
*TIMP3*	12.51	13.69	13.29	X	X		
*IL1RN*	11.06	8.60	10.47		X	X	

* HLA-DRA cells include immune regulatory and inflammatory macrophages, dendritic cells, activated proinflammatory fibroblasts and B cells.

## Data Availability

The data presented in this study are available on request from the corresponding author.

## Supplementary Materials

The following supporting information can be downloaded at: https://www.mdpi.com/article/10.3390/ijms25126450/s1.
